# A Genome-Wide Association Study on Calcium Nephrolithiasis in Chinese Han Population Identifies Novel Susceptible Loci at 4q35.1, 5q31.2 and 18q21.2

**DOI:** 10.3390/genes17030313

**Published:** 2026-03-10

**Authors:** Lujia Wang, Zijian Zhou, Xiaoling Lin, Kangcheng Luo, Peng Gao, Deke Jiang, Zhong Wu

**Affiliations:** 1 Department of Urology, Huashan Hospital & Institute of Urology, Fudan University, 12 Middle Wulumuqi Rd., Shanghai 200040, China; lukewang2006@126.com (L.W.); zjzhou21@m.fudan.edu.cn (Z.Z.); gaopeng76@163.com (P.G.); 2 Clinical Research Center of Urolithiasis, Shanghai Medical College, Fudan University, Shanghai 200040, China; 3 Institute of Nephrology, Department of Nephrology, Ruijin Hospital, Shanghai Jiao Tong University School of Medicine, Shanghai 200040, China; lxling.85@163.com; 4 The First School of Clinical Medicine, Nanjing Medical University, Nanjing 211166, China; luokangcheng@gmail.com; 5 State Key Laboratory of Organ Failure Research, Guangdong Key Laboratory of Viral Hepatitis Research, Department of Infectious Diseases and Hepatology Unit, Institutes of Liver Diseases Research of Guangdong Province, Nanfang Hospital, Southern Medical University, Guangzhou 510515, China; 6 Key Laboratory of Molecular Pathology in Tumors of Guangxi Higher Education Institutions, Department of Pathology, The Affiliated Hospital of Youjiang Medical University for Nationalities, Baise 533000, China

**Keywords:** calcium nephrolithiasis, genome-wide association study, Chinese Han population, *SORBS2*, *CXXC5*, *MBD2*

## Abstract

**Background**: Nephrolithiasis is a significant global health and economic challenge, with an increasing prevalence and a high recurrence rate. However, there is limited knowledge regarding the potential associations between calcium nephrolithiasis risk and Chinese Han populations currently. **Methods**: To identify the genetic factors for calcium nephrolithiasis, we presented a genome-wide association study (GWAS) using a total of 1006 calcium nephrolithiasis cases and 1200 controls of Chinese Han ethnicity. Suggestive loci (*p* < 1.0 × 10^−6^) were replicated in 445 cases and 1008 controls. We also assessed the association of GWAS-level significant single-nucleotide polymorphisms (SNPs) with quantitative traits, including metabolic, kidney-related, and electrolyte traits. **Results**: Here we found three novel loci for calcium nephrolithiasis: *SORBS2* on 4q35.1 (rs3736194; *p* = 2.84 × 10^−13^, OR = 0.6279), *CXXC5* on 5q31.2 (rs356450; *p* = 6.09 × 10^−16^, OR = 2.0312), and *MBD2* on 18q21.21 (rs55826947; *p* = 6.29 × 10^−10^, OR = 0.6017). Subsequent analyses revealed the association of SNP rs3736194 with higher serum carbon dioxide (*p* = 0.04666), rs356450 with lower serum chloride (*p* = 0.02992), and rs55826947 with higher BMI (*p* = 0.03174), respectively. **Conclusions**: We performed the first GWAS on calcium nephrolithiasis in a Chinese Han population cohort and identified three novel susceptibility loci on 4q35.1, 5q31.2, and 18q21.2. Further research into the molecular mechanisms underlying these variations in nephrolithiasis is warranted.

## 1. Background

Nephrolithiasis constitutes a significant global health and economic burden, impacting over 15% of men and more than 5% of women by the age of 70, with the global prevalence estimated to be approximately 10.85% recently [1]. The overall prevalence rate of nephrolithiasis was estimated to be 4.0% throughout mainland China [2]. Unfortunately, nephrolithiasis exhibits a high recurrence rate, with up to 50% of patients experiencing a subsequent episode within a decade following their initial onset [3]. The majority of kidney stones consist of either calcium oxalate or calcium phosphate crystals. The primary mechanism behind crystal formation and growth is the increased supersaturation of lithogenic salts in the urine, further exacerbated by the reduced urinary levels of natural stone inhibitors such as citrate, magnesium, pyrophosphate, and uromodulin [4]. The etiology of nephrolithiasis is complex, influenced by an interplay of environmental, lifestyle, and genetic factors. Studies indicate that approximately 65% of individuals with nephrolithiasis have a family history [5]. Furthermore, twin studies have demonstrated a significant genetic component, revealing a heritability estimate of over 45% [6]. Of particular note is the elevated risk observed in individuals with a substantial family history of urolithiasis; those with both a parent and a sibling affected exhibit a standard incidence ratio for developing stones that exceeds 50% [7]. This evidence underscores the substantial influence of genetic factors on the likelihood of nephrolithiasis, suggesting that both hereditary traits and environmental exposures play critical roles in the pathogenesis of kidney stones.

As far as we know, up to six genome-wide association studies (GWAS) of urolithiasis have been published, identifying 25 loci associated with nephrolithiasis [8,9,10,11,12,13]. The first GWAS in kidney stones, conducted in 2009 with Dutch and Icelandic cohorts, identified two synonymous variants in *CLDN14*. *CLDN14* is expressed in the kidney and plays a crucial role in regulating paracellular permeability at epithelial tight junctions [8]. In 2011, a GWAS in nephrolithiasis was performed in a Japanese population, which was the first one for an East Asian population. They identified three novel susceptible loci at 5q35.3 (*SLC34A1*), 7p14.3 (*AQP1*), and 13q14.1 (*DGKH*) [10]. We replicated the association of seven single-nucleotide polymorphisms (SNPs) in these loci with urolithiasis in the Chinese Han population. Unfortunately, none of these SNPs previously identified were significantly associated with urolithiasis risk in the Chinese Han population [14]. In 2018, a large-scale GWAS of urolithiasis performed in a Japanese population revealed 14 significant loci, 9 of which were novel loci. They speculated that genetic factors influencing metabolic and crystallization pathways function in the development of urolithiasis [12]. The largest GWAS to date in nephrolithiasis was performed in 2019, comprising a trans-ethnic meta-analysis that included cohorts from the UK and Japan. They identified 20 loci of interest, 7 of which have not been identified previously. Of these, five loci, including *DGKD*, *DGKH*, *WDR72*, *GPIC1*, and *BCR*, are supposed to influence the CaSR signaling pathway [13].

In our previous studies, we replicated the association of the reported 28 SNPs in 24 loci with calcium nephrolithiasis in a Chinese Han population, and our findings elucidated the significance of genetic variation at only two of these loci (*WDR72* and *PDILT*) in calcium nephrolithiasis [15,16]. This low replication rate highlights the significant genetic heterogeneity of nephrolithiasis across different ancestries, suggesting that the genetic architecture identified in other populations may not be directly transferable to the Chinese Han population. Therefore, a population-specific GWAS on nephrolithiasis in the Chinese Han population is urgently warranted to delineate its unique genetic landscape. 

To further investigate the genetic factors that are related to the risk of calcium nephrolithiasis in Chinese Han population, we conducted a GWAS of calcium nephrolithiasis in the Chinese Han population.

## 2. Methods

### 2.1. Study Participants

Table 1 showed the characteristics of each cohort. The discovery stage of our GWAS included 1010 cases of nephrolithiasis and 1200 healthy controls. For the replication stage, an independent sample set was used, consisting of 445 cases and 1008 controls. The participants for the discovery and replication stages were recruited during distinct periods. The diagnosis of nephrolithiasis in patients from Huashan Hospital of Fudan University was confirmed by a clinician. Patients with uric acid, struvite, cystine, ammonium acid urate stones, or secondary nephrolithiasis caused by drugs, hyperparathyroidism, gout, or urinary deformity were excluded from the analysis. Control subjects were recruited from a routine health check-up cohort at the same institution. All controls had no self-reported history of urolithiasis and were confirmed stone-free by ultrasonography. All the participants provided informed consent, and this study was approved by the Huashan Institutional Review Board of Fudan University (HIRB).

### 2.2. Genotyping and Quality Control in the GWAS Discovery Stage

DNA samples were genotyped using the Illumina Asian Screening Array (ASA) Beadchip (Illumina Inc., San Diego, CA, USA), resulting in a total of 659,184 SNPs genotyped in 1010 patients with calcium nephrolithiasis. Control samples were genotyped using the Illumina OmniExpress BeadChips (Illumina Inc., San Diego, CA, USA) based on our previously published GWAS [17]. A standard quality control procedure was applied to select samples and SNPs for further analysis, with samples excluded for the following reasons: (i) a genotyping rate of less than 95%; (ii) duplicates or probable familial relationships (IBS > 0.99); and (iii) ambiguous gender. SNPs were removed if they met any of these criteria: (i) call rate below 95%; (ii) minor allele frequency (MAF) less than 0.01; or (iii) *p* value less than 1 × 10^−3^ in the Hardy–Weinberg Equilibrium (HWE) test among controls.

Imputation analysis was conducted using the IMPUTE software (version 2) [18], with the 1000 Genomes Project Han Chinese in Beijing (CHB) population as a reference. The threshold for the imputation information score was set at greater than 0.90. Following quality control procedures, the analysis proceeded with a dataset comprising 1006 cases and 1200 controls, encompassing 1,868,144.

### 2.3. SNP Selection and Genotyping in Replication Stage

A subset of SNPs independently associated with the risk of calcium nephrolithiasis was selected for replication based on the following criteria: (i) each locus with one SNP reaching *p* < 5 × 10^−8^ or (ii) each locus with minimum five SNPs reaching *p* < 1.0 × 10^−6^ for discovery samples, and (iii) linkage disequilibrium (LD) *r*^2^ < 0.2 between markers. Genotyping analyses of replication for 445 case samples were conducted using the ASA BeadChip (Illumina Inc., San Diego, CA, USA). Genotyping for 1008 control samples was performed using the Illumina Human OmniExpress BeadChip in our previously published GWAS [19], which was conducted in a male cohort; consequently, all controls were male. To account for this sex imbalance, sex was included as a covariate in the logistic regression models for the replication analysis. All SNPs satisfied a call rate > 98% and had no deviation from HWE (*p* ≥ 0.001) in the controls.

### 2.4. Quantitative Trait Locus Analysis

The GWAS-level significant SNPs were used to assess the association with blood parameters, including metabolic traits (body mass index (BMI), total cholesterol, triglycerides, serum high-density lipoprotein (HDL), low-density lipoprotein (LDL), albumin and blood glucose), kidney-related traits (serum urea, urea, uric acid, estimated glomerular filtration rate (eGFR) and urine pH), and electrolytes traits (serum sodium, potassium, chloride, CO_2_, calcium, magnesium, phosphorus, alkaline phosphatase (ALP), parathyroid hormone (PTH) and calcifediol) through quantitative trait locus (QTL) analysis. Laboratory measurements were conducted within two weeks prior to surgery.

### 2.5. Statistical Analysis

The logistic regression model was utilized to assess the relationship between SNPs and the risk of calcium nephrolithiasis, based on an additive genetic model, employing PLINK version 1.07. Odds ratios (ORs) and 95% confidence intervals (CIs) were estimated with adjustment for the age, gender, and the top two principal components to control for potential population stratification. Principal component analysis (PCA), conducted using the PLINK software package (version 1.07), accounted for population stratification and sample quality control, with PCA results visualized through the R statistical program [20]. The results obtained from both the initial GWAS and the subsequent replication phase were integrated through a comprehensive meta-analysis. To evaluate the consistency of these findings across studies, we employed Cochran’s *Q* statistic and the *I*^2^ statistic to quantify the extent of variation across studies that could be attributed to heterogeneity rather than random chance. For the meta-analysis, a fixed-effects model (Mantel–Haenszel method) was applied for SNPs with *I*^2^ ≤ 50%, while a random-effects model (DerSimonian and Laird method) was used for SNPs with *I*^2^ > 50%. We established a stringent genome-wide significance threshold of *p* < 5.0 × 10^−8^, while in the replication stage, significance was determined post-Bonferroni correction at *p* < 3.33 × 10^−3^ (0.05/15). Regional plots were generated using LocusZoom [21], and data analysis was conducted using SAS 9.2 (SAS Institute, Cary, NC, USA) and R 4.3.3.

## 3. Results

### 3.1. Discovery Phase

We performed a multistage GWAS of calcium nephrolithiasis in the Han Chinese population, with a total of 1451 calcium nephrolithiasis cases and 2208 controls. The characteristics of the subjects in each stage are summarized in Table 1.

**Figure 1:**
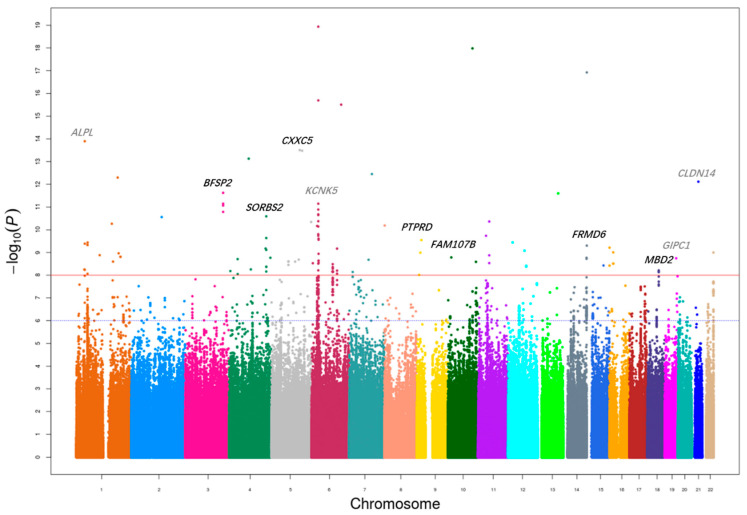
Manhattan plot of the genetic evidence of association for calcium nephrolithiasis in Chinese Han population using log-additive model. The *x*-axis shows the chromosomal position, and the *y*-axis represents the −log_10_ *p* additive value. The horizontal dashed blue line indicates the preset threshold of *p* = 5 × 10^−8^.

In the first discovery stage, 1,868,144 SNPs were genotyped using the Illumina ASA Beadchips in a cohort of 1010 cases and 1200 controls. After quality control filtering, 1,837,032 SNPs remained for analysis across 1006 cases and 1200 controls. Association analyses were conducted using logistic regression adjusting for age and gender as covariates, with the results illustrated in the Manhattan plots shown in Figure 1. PCA results did not indicate substantial stratification within the study population, and the quantile-quantile (Q-Q) plot analysis revealed a genomic inflation factor (λ) value of 1.6006 (Supplementary Figure S1). To further evaluate the source of this inflation, we performed LD score regression analysis, which yielded an intercept of 1.07, indicating that the observed inflation is largely attributable to polygenic architecture rather than confounding biases such as population stratification. In the discovery stage, 827 SNPs had an association *p* value < 1.0 × 10^−6^, and 241 SNPs attained the genome-wide significance (*p* < 5.0 × 10^−8^) (Supplementary Table S1). Additionally, we evaluated the performance of previously identified SNPs related to nephrolithiasis risk: out of the 42 reported SNPs, 12 were included in our discovery phase, with 3 achieving statistical significance (Supplementary Table S2).

### 3.2. Replication Phase

**Figure 2:**
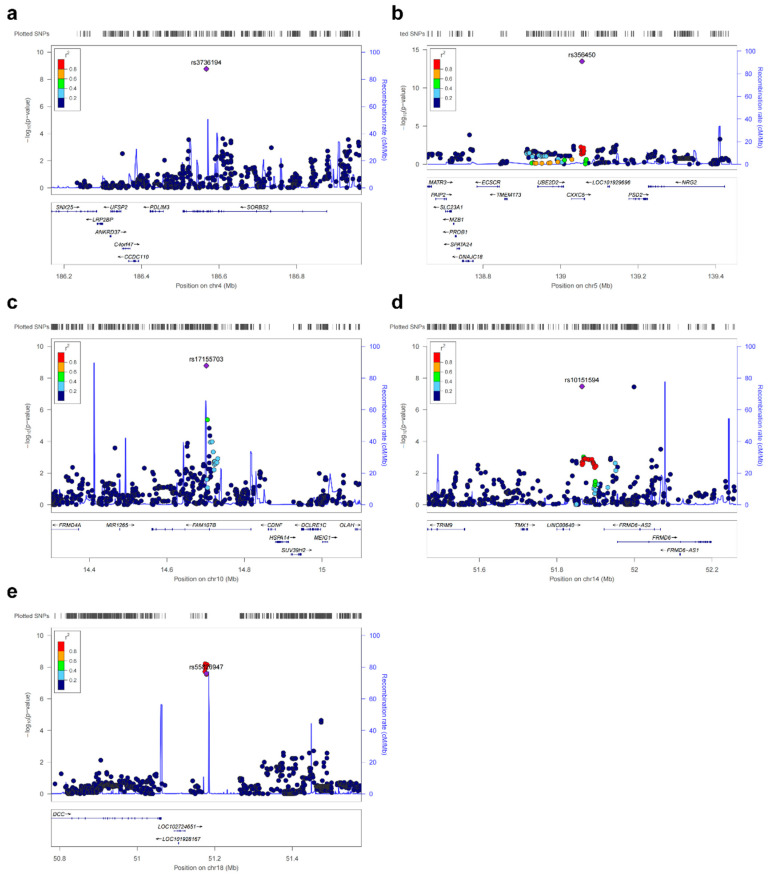
Regional plots of association results for the newly identified susceptibility loci for calcium nephrolithiasis. Regional plots at (**a**) 4q35.1, (**b**) 5q31.2, (**c**) 10p13, (**d**) 14q22.1 and (**e**) 18q21.2. Results (−log_10_*p*) are shown for SNPs in the regions flanking 500 kb on either side of the index SNPs (purple diamond). The index SNPs are shown as diamonds, and the *r*^2^ values of the remaining SNPs are indicated by color. The genes within the region are annotated and indicated by arrows.

To validate the suggestive associations identified during the discovery stage, we selected SNPs that were independently associated with the condition for a validation study. This subsequent phase involved an additional 445 cases and 1008 controls, all of whom were from the Chinese Han population. We selected 15 SNPs in 15 genomic regions with significant associations using the thresholds of *p* < 1.0 × 10^−6^ and *r*^2^ < 0.2, and adjusted for lead SNPs in each region. Five loci on chromosomes 4, 5, 10, 14 and 18 were successfully replicated: rs3736194 at 4q35.1 (*p* = 3.18 × 10^−5^), rs356450 at 5q31.2 (*p* = 3.87 × 10^−4^), rs17155703 at 10p13 (*p* = 5.85 × 10^−4^), rs10151594 at 14q22.1 (*p* = 5.60 × 10^−4^), and rs55826947 at (*p* = 3.11 × 10^−3^) (Figure 2a–e, Supplementary Table S3).

### 3.3. Combined Analysis

Employing a meta-analysis to integrate the discovery and replication stage, three novel SNPs, rs3736194 at 4q35.1 (OR = 0.6279, *P*_meta_ = 2.84 × 10^−13^), rs356450 at 5q31.2 (OR = 2.0312, *P*_meta_ = 6.09 × 10^−16^), and rs55826947 at (OR = 0.6017, *P*_meta_ = 6.29 × 10^−10^), exceeded the genome-wide significant threshold (Table 2). However, rs17155703 in *FAM107B* at 10p13 (OR = 1.0503, *P*_meta_ = 0.9385) and rs10151594 in *FRMD6* at 14q22.1 (OR = 1.0545, *P*_meta_ = 0.9214) failed to reach the genome-wide significance. No evidence for heterogeneity was found among the two stages at rs3736194 (*P*_het_ = 0.7457) and rs55826947 (*P*_het_ = 0.2246).

### 3.4. Quantitative Trait Locus Analysis

To further investigate the roles of SNPs in the pathogenesis of calcium nephrolithiasis, we explored the associations between five genetic variants and 22 quantitative traits across three categories: metabolic, kidney-related, and electrolyte (Table 3). Notably, the G allele of rs3736194 was significantly linked to increased serum carbon dioxide levels (*p* = 0.04666), while the G allele of rs356450 was associated with lower serum chloride levels (*p* = 0.02992). Additionally, the T allele of rs55826947 correlated with a higher BMI (*p* = 0.03174) and showed a suggestive association with decreased serum alkaline phosphatase levels (*p* = 0.05634). The C allele of rs17155703 was significantly associated with reduced serum albumin levels (*p* = 0.04223), and the A allele of rs10151594 exhibited a suggestive association with elevated serum albumin levels (*p* = 0.05809).

## 4. Discussion

To our knowledge, there has been no GWAS on the potential genetic association with the risk of calcium nephrolithiasis in the Chinese Han population. By conducting a two-stage GWAS analysis with 1451 calcium nephrolithiasis cases and 2208 controls, we identified three novel loci on 4q35.1, 5q31.2, and 18q21.2, which were associated with calcium nephrolithiasis.

rs3736194 on 4q35.1 is an intronic variant in *SORBS2*, encoding Sorbin and SH3 Domain Containing 2, which is a key number of the sorbin homology family of adapter and scaffold proteins. In the glomeruli of kidney, *SORBS2* is involved in the formation of stress fibers in the glomeruli, which is suggested to be associated with the development of diabetic nephropathy [22]. Beyond its role in glomerular pathology, *SORBS2* may contribute to nephrolithiasis through mechanisms involving crystal formation and handling. In patients with coronary atherosclerosis, *SORBS2* is substantiated to regulate lipid-induced inflammation and foam cell formation [23]. This is particularly relevant because medullary macrophages in the kidney are known to monitor and remove crystal particles from the tubular fluid and interstitium, playing a crucial role in preventing stone retention [24]. Therefore, we speculate that *SORBS2* variants could influence the inflammatory phenotype or phagocytic capacity of these renal macrophages, thereby affecting crystal clearance and promoting nephrolithiasis. In our study, the risk allele of rs3736194 was found to be significantly associated with elevated serum carbon dioxide levels. This finding underscores a potential genetic link between this specific allele and altered metabolic processes affecting carbon dioxide in the bloodstream. It has been reported that the risk of calcium phosphate stones increased in patients with higher level of serum CO_2_ [25]. Our findings indicate that the variation in *SORBS2* might increase the risk of calcium nephrolithiasis by influencing abnormal serum CO_2_ metabolism.

rs356450 is an intronic variant in *CXXC5* on 5q31.2, encoding a retinoid-inducible nuclear factor (RINF), and is a member of the CXXC-type zinc-finger protein family. *CXXC5* is highly expressed in adipose tissue of obese patients with type 2 diabetes. The variation in *CXXC5* expression has been reported to be resistant to obesity-related metabolic diseases [26]. Most notably, *CXXC5* has been identified as a vitamin D receptor (VDR) interacting protein, which is a potent and dose-dependent activator of VDR/vitamin D responsive element-mediated transcription [27]. VDR is a master regulator of calcium homeostasis, directly controlling the expression of genes involved in intestinal calcium absorption and renal calcium transport. Polymorphisms in the *VDR* itself have been consistently associated with calcium nephrolithiasis and disorders of bone mineral density. It has been reported that *CXXC5* knockout mice exhibit high bone mass phenotypes, with significantly increased bone thickness and bone volume density [28]. Since the variation in the *VDR* gene is associated with diseases of abnormal calcium metabolism, such as mineral density regulation and the development of calcium nephrolithiasis, we propose that the association of *CXXC5* with nephrolithiasis is mediated through its regulatory effects on *VDR* signaling, leading to altered calcium homeostasis and increased stone risk.

rs55826947 on 18q21.2 is an upstream transcript variant of *MBD2*, a gene encoding Methyl-CpG-binding domain protein 2 (MBD2), a protein reader of methylation. Recent studies have demonstrated that the inhibition of *MBD2* can effectively mitigate the progression of renal fibrosis induced by unilateral ureteral obstruction and ischemia/reperfusion [29]. Moreover, the *MBD2* gene is believed to be involved in obesity and insulin resistance [30]. Insulin resistance, a core feature of obesity, leads to impaired renal ammonia production and low urine pH, which promotes the crystallization of calcium oxalate and uric acid. In our study, the variation in *MBD2* was associated with higher BMI. Epidemiologic studies have demonstrated that stone risk incidence increases with BMI, a well-established risk factor for stone formation. Given the epidemiological link between increasing BMI and stone risk [31], we speculated that the variance in *MBD2* might increase the risk of calcium nephrolithiasis by increasing by body weight.

We observed a suggestive association of rs17155703 on 10p13 with calcium nephrolithiasis. rs17155703 is an intronic variant in *FAM107B*, encoding Family with sequence similarity 107 member B (FAM107B) protein, an 18 kDa nuclear protein belonging to the Family with sequence similarity 107, also known as heat shock-inducible tumor small protein (HITS) [32]. The role of the gene *FAM107B* in the pathogenesis of nephrolithiasis remains unclear. Further research is required to determine how this gene may contribute to stone formation in the kidneys. Additionally, the chromosomal region 10p13 has been identified as a susceptibility locus for type 2 diabetes mellitus through GWASs conducted in both European and Asian populations [33,34].

In the present study, rs10151594 on 14q22.1 showed a suggestive association and is an upstream transcript variant of FRMD6, which encodes the FERM Domain Containing 6 (FRMD6). This protein serves as a key activator of the Hippo kinase pathway, playing crucial roles in regulating cell proliferation and apoptosis [35]. *FRMD6* has been implicated in a range of complex diseases, including asthma, aortic aneurysms, Alzheimer’s disease, and some cancers [36,37]. Additionally, it has been reported that the variation in *FRMD6* in immune cells could impact the binding properties of *VDR* to its receptor elements within *FRMD6*, potentially influencing the transcriptional response of FRMD6 to 1,25-dihydroxyvitamin D3 [38].

Several limitations of this study should be acknowledged. First, the sample size in the initial GWAS discovery stage was insufficient to identify all potential genetic susceptibility loci associated with calcium nephrolithiasis. Second, the control samples in the replication stages were genotyped using a different platform. This cross-platform design may have introduced technical variability, potentially affecting the consistency of the replication signals. Finally, the biological interpretation of the identified loci remains largely speculative and is based on indirect evidence. Further experimental studies, including functional assays and animal models, are warranted to elucidate the precise mechanisms by which these genes may contribute to the pathogenesis of calcium nephrolithiasis.

## 5. Conclusions

In conclusion, we conducted a GWAS of calcium nephrolithiasis in the Chinese Han population for the first time, identifying three novel susceptibility loci located on 4q35.1, 5q31.2, and 18q21.2. While further investigation is needed to elucidate the molecular mechanisms by which these variations may elevate the risk of nephrolithiasis, these findings indicate that factors such as calcium homeostasis, renal function, and metabolism could play significant roles in the development of calcium nephrolithiasis.

## Figures and Tables

**Table 1:** Characteristics of samples and methods used in this study.

Stage	Number of Samples		Female (%)			Age (Mean ± SD, in Years)		
	Cases	Controls	Cases	Controls	*p*	Cases	Controls	*p*
Discovery	1006	1200	31.81	37.67	0.0041	52.97 ± 13.22	51.08 ± 9.75	0.00017
Replication	445	1008	33.26	0	-	53.33 ± 14.44	49.98 ± 12.65	0.038
QTL analysis	1451	-	32.25	-	-	53.08 ± 13.60	-	-

**Table 2:** The result of association analysis of nephrolithiasis in each stage.

Chr	SNP	EA	Position	Genes in the Region	Stage 1				Stage 2				Combined			
					Case EAF	Control EAF	*p*	OR	Case EAF	Control EAF	*p*	OR	*P*_meta_	OR	*Q*	*I* ^2^
4	rs3736194	G	186566987	SORBS2	0.1540062	0.2275	1.71 × 10^−9^	0.618142006	0.1667	0.2366	3.18 × 10^−5^	0.6453	**2.84 × 10^−13^**	0.6279	0.7457	0
5	rs356450	G	139055009	CXXC5	0.1149254	0.051926298	3.33 × 10^−14^	2.370777349	0.1112	0.07164	0.000387	1.622	**6.09 × 10^−16^**	2.0312	0.0333	77.92
10	rs17155703	C	14701678	FAM107B	0.0623722	0.115966387	1.65 × 10^−9^	1.97198155	0.05012	0.0873	0.000585	1.813	0.9385	1.0503	0	97.33
14	rs10151594	A	51864477	LINC00640, FRMD6	0.0746347	0.126672241	3.37 × 10^−8^	1.798361045	0.0808	0.1254	0.00056	1.631	0.9214	1.0545	0	97.26
18	rs55826947	T	51178305	LOC102724651, MBD2	0.0743927	0.12649063	2.52 × 10^−8^	0.555025377	0.09908	0.1391	0.003105	0.6807	**6.29 × 10^−10^**	0.6017	0.2246	32.2

Chr, chromosome; Position, Based on NCBI Genome Build 37 (hg19); EA, effect allele; EAF, effect allele frequency; OR, odds ratio, calculated for the effect allele. The bold ***P***_meta_ indicates statistical significance at the genome-wide level.

**Table 3:** Multiple linear regression analyses for clinical parameters.

	rs3736194			rs356450			rs17155703			rs10151594			rs55826947		
	Beta ^a^	S.E. ^b^	*p*	Beta	S.E.	*p*	Beta	S.E.	*p*	Beta	S.E.	*p*	Beta	S.E.	*p*
BMI	0.3965	0.3149	0.2083	−0.5618	0.3651	0.1242	−0.04364	0.4737	0.9266	−0.2839	0.4513	0.5295	0.8835	0.4108	**0.03174**
Serum TC	−0.004285	0.2272	0.985	−0.1202	0.2502	0.6321	0.2332	0.3873	0.5487	0.0571	0.3436	0.8684	0.04876	0.3827	0.8989
Serum TG	−0.1649	0.1563	0.2941	0.2293	0.1718	0.1855	−0.2262	0.2722	0.408	−0.1185	0.2358	0.6166	−0.103	0.2658	0.6994
Serum HDL	0.03081	0.04251	0.4707	0.02173	0.04665	0.6426	0.07966	0.07237	0.2742	0.03705	0.06594	0.5758	−0.05088	0.07352	0.4908
Serum LDL	0.4445	0.521	0.3959	−0.5984	0.5661	0.2933	−0.3262	0.8948	0.7164	−0.2594	0.8002	0.7467	−0.119	0.9083	0.8961
Blood glucose	−0.08921	0.07497	0.2344	−0.01402	0.08803	0.8735	0.01878	0.1138	0.8689	−0.03709	0.1055	0.7253	0.1249	0.09908	0.2078
Serum albumin	0.1767	0.2524	0.484	−0.09845	0.2961	0.7396	−0.7772	0.3821	**0.04223**	0.6859	0.3615	0.05809	−0.2569	0.3325	0.4399
Serum urea	−0.04317	0.1721	0.802	0.2887	0.1991	0.1473	0.108	0.2587	0.6765	−0.1659	0.2457	0.4997	−0.1436	0.2217	0.5174
Serum creatinine	2.27	2.348	0.3339	−0.3888	2.729	0.8868	−1.899	3.533	0.5912	0.2215	3.366	0.9476	−3.81	3.051	0.212
eGFR ^c^	−0.4085	1.566	0.7943	−1.293	1.852	0.4853	2.743	2.387	0.2509	−2.342	2.242	0.2963	1.4	2.071	0.4991
Serum uric acid	0.002351	0.007124	0.7414	0.001215	0.008355	0.8844	−0.01294	0.01082	0.2317	0.00187	0.0103	0.856	-0.005093	0.00935	0.5861
Urine pH	0.05404	0.04191	0.1976	−0.01142	0.04911	0.8162	−0.06176	0.06315	0.3283	−0.02063	0.06012	0.7316	−0.03138	0.05507	0.5689
Serum sodium	−1.622	2.596	0.5322	−1.624	2.986	0.5867	−1.086	3.894	0.7803	−0.9745	3.731	0.794	−1.242	3.376	0.7131
Serum potassium	−0.09068	0.1081	0.4019	−0.09901	0.1246	0.4269	−0.07535	0.1624	0.6428	−0.06079	0.1554	0.6957	−0.09661	0.1408	0.4928
Serum chloride	0.1682	0.4576	0.7133	−1.498	0.6881	**0.02992**	−0.9178	0.9419	0.3304	−0.9573	0.8962	0.286	−1.103	0.7774	0.1566
Serum CO_2_	0.3328	0.1671	**0.04666**	−0.2824	0.1955	0.1489	0.02982	0.2521	0.9058	−0.0616	0.238	0.7958	0.004017	0.2169	0.9852
Serum calcium	0.009204	0.007819	0.2394	0.003338	0.009128	0.7147	−0.009693	0.01174	0.4094	0.01204	0.01116	0.2808	0.01059	0.01034	0.3058
Serum magnesium	−0.02568	0.05017	0.6089	−0.02901	0.05762	0.6148	−0.02318	0.07542	0.7587	−0.0208	0.07147	0.7711	−0.03205	0.06504	0.6223
Serum phosphorus	−0.003505	0.01258	0.7807	0.02518	0.01464	0.08578	−0.0137	0.01886	0.468	−0.005565	0.01789	0.7559	0.01339	0.01652	0.418
Serum ALP	−1.066	1.566	0.4961	1.938	1.854	0.2961	−3.064	2.395	0.2011	0.432	2.276	0.8495	−3.961	2.073	0.05634
Serum PTH	−2.336	6.267	0.71	−6.081	6.383	0.3426	9.484	8.15	0.247	5.132	7.738	0.5086	−2.93	7.469	0.6956
Serum calcifediol	5.081	3.819	0.1873	−1.502	3.987	0.7074	−8.091	5.887	0.1732	−0.9936	5.009	0.8433	−0.5836	3.283	0.8594

^a^ Beta: regression coefficient. ^b^ S.E.: standard error of mean. ^c^ eGFR (mL/min/1.73 m^2^) = 186 × (serum creatinine/88.41) − 1.154 × age − 0.203 (×0.742 if female). Footnote: BMI: Body mass index, TC: Total cholesterol, TG: Triglyceride, HDL: High-density lipoprotein, LDL: Low-density lipoprotein, eGFR: Estimated glomerular filtration rate, ALP: alkaline phosphatase, PTH: parathyroid hormone. The bold means *p* < 0.05.

## Data Availability

The original contributions presented in the study are included in the article/Supplementary Materials. Further inquiries can be directed to the corresponding authors.

## Supplementary Materials

The following supporting information can be downloaded at: https://www.mdpi.com/article/10.3390/genes17030313/s1, Figure S1: The quantile–quantile (Q-Q) plot of expected *p* values versus observed *p* values in calcium nephrolithiasis. Supplementary Table S1: SNPs with *p*-value of less than 5 × 10^−8^ in discovery stage. Supplementary Table S2: Association results in GWAS stage for Nephrolithiasis susceptibility loci identified in populations of European or Japanese populations. Supplementary Table S3: The result of association analysis of nephrolithiasis in the replication stage.

## Abbreviations

CI: Confidence interval; QTL: Quantitative trait loci; GWAS: Genome-wide association study; LD: Linkage disequilibrium; MAF: Minor allele frequency; SNP: Single-nucleotide polymorphism.
