# Supplementary material for: A Genome-Wide Association Study on Calcium Nephrolithiasis in Chinese Han Population Identifies Novel Susceptible Loci at 4q35.1, 5q31.2 and 18q21.2
Source: Genes (Basel). 2026 Mar 10;17(3):313. doi: 10.3390/genes17030313 (PMC13026470; doi:10.3390/genes17030313)
Supplement: Supplementary file 1 [file genes-17-00313-s001.zip › genes-4178882-supplementary.pdf]

Supplementary Figure S1. The quantile-quantile (Q-Q) plot of expected *P* values versus observed *P* values in calcium nephrolithiasis.

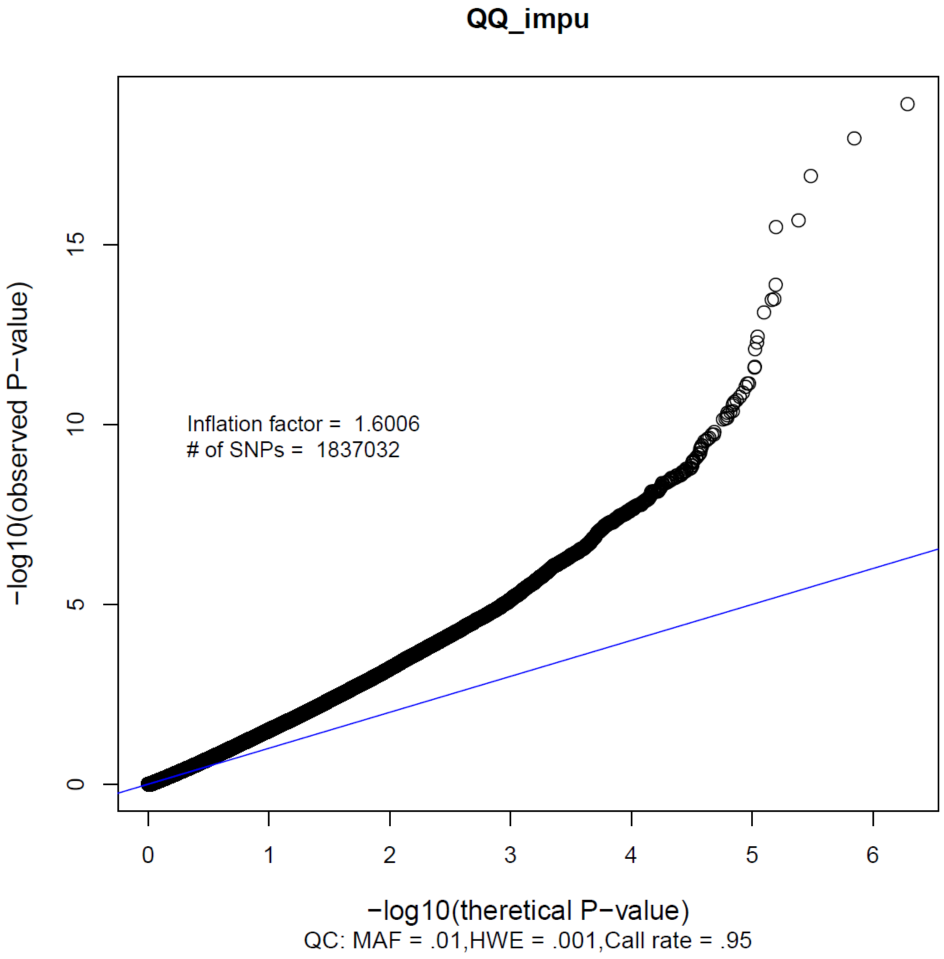

Supplementary Table S1. SNPs with *P*-value of less than 5×10^{-8} in discovery stage.

CHR	SNP	BP	Allele1	Allele2	Control Allele1	Control Allele2	Case Allele1	Case Allele2	ControlAllele1 Freq	ControlAllele2 Freq	HWE	OR	P
10	rs1119161	71367438	G	T	1598	792	1783	159	0.668619247	0.331380753	0.254549899	0.179927429	2.45E-86
13	rs537909	72811874	C	T	1533	851	1749	221	0.643036913	0.356963087	0.838795917	0.227622432	1.98E-77
7	rs11762974	16887396	A	G	224	2162	595	1377	0.093880972	0.906119028	0.737360598	0.239777983	3.90E-68
5	rs10079526	124025160	A	C	838	1550	251	1721	0.350921273	0.649078727	0.481821104	3.706973397	2.14E-64
2	rs10199464	149080548	A	G	2165	133	1564	438	0.942123586	0.057876414	0.011852632	4.558727381	6.03E-54
2	rs1669518	106620083	C	T	1210	1112	1457	549	0.521102498	0.478897502	0.744871817	0.410008987	2.14E-43
1	rs4657441	165427882	C	T	1073	1303	516	1494	0.451599327	0.548400673	0.280247913	2.384274215	1.23E-40
10	rs11200592	124138947	C	T	2026	326	1356	584	0.861394558	0.138605442	0.339923799	2.676547768	3.51E-38
6	rs6905926	19610320	A	G	657	1687	319	1689	0.280290102	0.719709898	0.282328757	2.062002813	1.46E-21
6	rs9264533	31234494	C	T	2080	264	1536	420	0.887372014	0.112627986	0.441550456	2.154356061	1.15E-19
10	rs4918354	110126606	A	G	402	1898	579	1429	0.174782609	0.825217391	0.590599884	0.522737324	1.04E-18
14	rs79570117	107178254	C	T	362	1978	137	1815	0.154700855	0.845299145	0.576090932	2.4245902	1.19E-17
6	rs116765566	31299083	C	T	1872	434	1416	594	0.811795317	0.188204683	0.369098287	1.809419667	2.03E-16
6	rs59276129	134743293	G	T	1821	575	1659	271	0.760016694	0.239983306	0.811596467	0.517326834	3.11E-16
1	rs1984985	38662594	A	G	194	2116	312	1620	0.083982684	0.916017316	0.311451731	0.476043333	1.27E-14
5	rs13166277	128284315	C	T	335	1947	146	1856	0.146801052	0.853198948	0.621446088	2.187277934	3.13E-14
5	rs356450	139055009	A	G	2264	124	1779	231	0.948073702	0.051926298	0.868996331	2.370777349	3.33E-14
4	rs7689871	88775875	A	G	1372	968	1374	598	0.586324786	0.413675214	0.418029968	0.616869369	7.43E-14
7	rs62483497	102332238	A	G	1467	927	1442	568	0.612781955	0.387218045	0.466676296	0.62335214	3.54E-13
1	rs964665	187209952	A	G	2070	328	1791	131	0.863219349	0.136780651	0.612988325	0.461606815	5.05E-13
21	rs4817237	30024756	C	T	1400	984	1382	614	0.587248322	0.412751678	0.081595041	0.63211088	7.65E-13
3	rs11921771	171119849	C	T	295	2003	419	1591	0.128372498	0.871627502	0.520881901	0.559238708	2.36E-12
13	rs4773679	92994893	C	T	1484	916	1384	538	0.618333333	0.381666667	0.686162423	0.629773834	2.51E-12

3	rs4894599	171119365	A	G	2007	295	1589	413	0.871850565	0.128149435	0.526276221	1.768281938	7.07E-12
6	rs9264483	31232446	C	T	2103	265	1630	372	0.888091216	0.111908784	0.434589014	1.811126288	7.12E-12
3	rs9862688	171123796	C	T	290	2008	404	1578	0.126196693	0.873803307	0.630399486	0.564104966	8.51E-12
6	rs9264482	31232412	G	T	2108	268	1630	372	0.887205387	0.112794613	0.489180887	1.795110338	1.29E-11
3	rs1985110	171121196	A	G	2012	294	1579	405	0.872506505	0.127493495	0.552887587	1.755308837	1.63E-11
6	rs9264492	31232676	G	T	2089	267	1630	372	0.886672326	0.113327674	0.491825245	1.785593162	2.11E-11
6	rs9264493	31232697	A	G	263	2061	372	1630	0.113166954	0.886833046	0.444025914	0.559142394	2.25E-11
4	rs6840755	168073668	A	G	2204	124	1792	218	0.946735395	0.053264605	0.063087717	2.162262385	2.56E-11
2	rs7601268	136790020	G	T	1392	946	1393	617	0.595380667	0.404619333	0.637163951	0.651751661	2.76E-11
6	rs9264554	31234855	C	T	2087	269	1637	373	0.88582343	0.11417657	0.536961863	1.76778857	4.21E-11
11	rs148834771	50602029	C	T	362	1970	178	1832	0.155231561	0.844768439	0.893952836	1.891245081	4.33E-11
5	rs4588548	179803397	C	T	254	2046	362	1632	0.110434783	0.889565217	0.296936351	0.55967985	4.52E-11
1	rs113622460	160870182	G	T	2209	191	1722	284	0.920416667	0.079583333	0.124319036	1.907425312	5.43E-11
8	rs185050949	1122397	A	G	188	2112	291	1717	0.08173913	0.91826087	0.268491782	0.525219983	6.49E-11
6	rs989134	26336224	A	C	198	2104	302	1708	0.086012163	0.913987837	0.99545156	0.532231259	6.82E-11
6	rs9264555	31234911	A	G	268	2086	369	1633	0.113848768	0.886151232	0.51671977	0.568565245	7.19E-11
6	rs9264454	31231332	A	G	2047	267	1628	370	0.884615385	0.115384615	0.372515564	1.742424242	1.57E-10
11	rs58776186	36007447	A	G	243	2051	337	1599	0.105928509	0.894071491	0.669217906	0.562159011	1.84E-10
6	rs9264548	31234771	A	G	280	2120	373	1637	0.116666667	0.883333333	0.743817617	0.579644899	1.84E-10
4	rs10005736	168074664	A	G	125	2199	212	1786	0.053786575	0.946213425	0.070454085	0.478884055	2.31E-10
6	rs9272678	32608915	C	T	1616	758	1133	797	0.680707666	0.319292334	0.840001469	1.499686777	2.46E-10
6	rs28374650	32623367	C	T	1874	428	1474	536	0.814074718	0.185925282	0.655906641	1.592183517	2.76E-10
9	rs73648215	20464479	C	T	1896	504	1735	275	0.79	0.21	0.441831374	0.596267325	2.84E-10
12	rs1604542	21181226	A	C	1633	653	1254	756	0.714348206	0.285651794	0.294317777	1.50763865	3.62E-10
1	rs9436458	51457656	G	T	1995	293	1610	400	0.871940559	0.128059441	0.209522563	1.691645645	3.63E-10
1	rs75192626	38650079	A	G	154	2178	244	1766	0.066037736	0.933962264	0.781223434	0.511756913	4.09E-10

1	rs11585823	50865101	A	G	263	2021	356	1582	0.115148862	0.884851138	0.329445087	0.578290311	4.64E-10
1	rs76910682	50874220	A	G	263	2021	356	1582	0.115148862	0.884851138	0.329445087	0.578290311	4.64E-10
14	rs8007269	107181426	C	T	1365	967	986	1024	0.585334477	0.414665523	0.545583006	1.465983962	4.98E-10
16	rs3176373	128054	A	G	120	2164	204	1768	0.052539405	0.947460595	0.698162057	0.480591497	6.14E-10
4	rs114399340	164961243	C	T	416	1978	205	1713	0.173767753	0.826232247	0.38048606	1.757399689	6.71E-10
6	rs143618813	116064559	C	T	282	2010	382	1608	0.123036649	0.876963351	0.025630997	0.590575916	6.74E-10
4	rs17052975	168047325	A	G	2214	124	1750	202	0.946963216	0.053036784	0.197749448	2.060958525	7.67E-10
12	rs11610146	74824368	C	T	161	2199	247	1763	0.068220339	0.931779661	0.361692316	0.522583876	8.34E-10
16	rs62043525	16884729	A	G	261	2139	110	1844	0.10875	0.89125	0.696825143	2.045492796	9.87E-10
22	rs135793	49946905	A	G	405	1905	211	1731	0.175324675	0.824675325	0.683509997	1.744113147	1.00E-09
9	rs140851803	15903310	C	T	2158	124	1752	206	0.9456617	0.0543383	1	2.046269701	1.02E-09
1	rs10047097	191586713	C	T	2178	122	1725	199	0.946956522	0.053043478	0.876671234	2.059501069	1.09E-09
6	rs68033958	32634226	A	G	367	1983	456	1532	0.156170213	0.843829787	0.484594664	0.62178075	1.14E-09
1	rs190700306	105904640	G	T	212	2082	302	1664	0.092414996	0.907585004	0.418933051	0.561049933	1.31E-09
11	rs116881177	50490474	C	T	339	1955	173	1815	0.147776809	0.852223191	0.897736881	1.819212631	1.33E-09
1	rs10919750	199833556	C	T	134	2200	217	1793	0.057412168	0.942587832	0.851273635	0.503271889	1.55E-09
1	rs10919751	199833564	C	T	2200	134	1793	217	0.942587832	0.057412168	0.851273635	1.986997528	1.55E-09
10	rs17155703	14701678	C	T	276	2104	122	1834	0.115966387	0.884033613	0.480211788	1.97198155	1.65E-09
4	rs3736194	186566987	A	G	1854	546	1626	296	0.7725	0.2275	0.470596911	0.618142006	1.71E-09
14	rs117089735	106006798	A	G	599	1723	688	1322	0.25796727	0.74203273	0.787140904	0.668012458	1.71E-09
19	rs11084078	51906317	A	G	1922	364	1543	467	0.840769904	0.159230096	0.736979434	1.59809633	1.80E-09
14	rs79557891	105998221	C	T	1727	575	1340	670	0.750217202	0.249782798	0.836433779	1.50173913	1.90E-09
4	rs148423240	38851450	A	G	184	2108	275	1725	0.080279232	0.919720768	0.212772425	0.547524582	1.95E-09
5	rs2561483	123637299	C	T	461	1939	239	1687	0.192083333	0.807916667	0.819776593	1.678186789	2.06E-09
7	rs138634107	87452322	G	T	149	2153	235	1767	0.064726325	0.935273675	0.519743884	0.520368412	2.09E-09
6	rs28636516	32606701	A	G	1934	348	1558	450	0.847502191	0.152497809	0.993672973	1.605174627	2.30E-09

6	rs28383322	32592796	C	T	1999	303	1609	401	0.868375326	0.131624674	0.358745015	1.644214577	2.31E-09
5	rs1396612	77121824	A	C	283	2117	366	1642	0.117916667	0.882083333	1	0.599732584	2.48E-09
5	rs1396613	77121794	C	T	2117	283	1642	366	0.882083333	0.117916667	1	1.667409821	2.48E-09
5	rs2909880	107408395	C	T	2171	163	1762	248	0.930162811	0.069837189	0.591248162	1.874640502	2.52E-09
1	rs1349526	166174460	C	T	1276	1112	1247	751	0.534338358	0.465661642	0.9450936	0.691065752	2.52E-09
10	rs10901840	125990522	A	G	1920	426	1702	222	0.818414322	0.181585678	0.252666183	0.587875077	2.58E-09
6	rs9274498	32633941	A	G	1935	361	1534	456	0.842770035	0.157229965	0.487102206	1.593357579	2.87E-09
11	rs117559791	50473035	A	G	336	1954	174	1818	0.146724891	0.853275109	0.971795876	1.796632902	2.93E-09
6	rs663536	6563646	A	G	367	1925	466	1540	0.160122164	0.839877836	0.846311428	0.630042918	2.96E-09
16	rs62043526	16886094	A	C	256	2138	110	1844	0.106934002	0.893065998	0.583311849	2.007245514	3.08E-09
16	rs62043527	16886108	G	T	2138	256	1844	110	0.893065998	0.106934002	0.583311849	0.49819516	3.08E-09
6	rs3094006	31461558	C	T	1997	325	1586	418	0.860034453	0.139965547	0.668630773	1.619450965	3.10E-09
6	rs62417683	96561591	A	G	301	2015	145	1815	0.129965458	0.870034542	0.444799523	1.869821169	3.26E-09
5	rs10942832	77120649	A	G	285	2115	367	1643	0.11875	0.88125	0.873292985	0.603262025	3.51E-09
15	rs61047849	75306985	A	G	125	2249	201	1809	0.052653749	0.947346251	0.481694418	0.500222321	3.72E-09
16	rs2541636	123220	A	G	2181	125	1793	205	0.945793582	0.054206418	0.948657202	1.994891244	3.78E-09
12	rs61934123	82613153	A	G	2126	182	1740	270	0.921143847	0.078856153	0.591543147	1.812618416	3.88E-09
12	rs61934124	82613171	G	T	2104	180	1740	270	0.921190893	0.078809107	0.868181112	1.813793103	4.18E-09
4	rs28465014	168055477	A	C	129	2217	203	1761	0.054987212	0.945012788	0.097026188	0.504762794	4.25E-09
4	rs7659299	168054033	C	T	2217	129	1761	203	0.945012788	0.054987212	0.097026188	1.981128587	4.25E-09
6	rs141362726	96570804	C	T	2092	306	1810	142	0.872393661	0.127606339	0.44005665	0.536352147	4.34E-09
6	rs28660974	96539970	C	T	307	2011	151	1821	0.13244176	0.86755824	0.57903499	1.841023378	4.74E-09
6	rs9390867	96564093	A	G	309	2091	145	1815	0.12875	0.87125	0.502097261	1.849750161	5.21E-09
4	rs138828367	98347642	C	T	120	2174	200	1810	0.052310375	0.947689625	0.329071785	0.499540018	5.55E-09
1	rs10796884	36858680	A	G	2092	308	1622	388	0.871666667	0.128333333	0.559170428	1.624769805	5.60E-09
1	rs16824718	38647366	A	G	117	2215	191	1789	0.050171527	0.949828473	0.377840589	0.494753761	5.79E-09

6	rs1140546	31322888	A	G	344	1982	434	1572	0.147893379	0.852106621	0.238825386	0.628662571	6.12E-09
18	rs7228258	51175484	A	G	300	2054	144	1826	0.127442651	0.872557349	0.25054058	1.852077248	6.14E-09
6	rs62416189	116100331	A	G	295	2003	379	1573	0.128372498	0.871627502	0.142305731	0.611266478	6.29E-09
4	rs73212615	5968519	A	G	2106	184	1722	270	0.919650655	0.080349345	0.965592043	1.794614452	6.57E-09
4	rs9968512	168052023	G	T	129	2195	203	1759	0.055507745	0.944492255	0.102307341	0.509242905	6.87E-09
18	rs1442081	51180280	G	T	2052	306	1842	150	0.870229008	0.129770992	0.347210741	0.546081625	6.90E-09
6	rs11156189	96547345	A	G	309	2091	146	1816	0.12875	0.87125	0.502097261	1.838092805	7.00E-09
6	rs11156196	96550527	G	T	309	2091	146	1816	0.12875	0.87125	0.502097261	1.838092805	7.00E-09
6	rs12210170	96549518	A	G	2091	309	1816	146	0.87125	0.12875	0.502097261	0.544042171	7.00E-09
6	rs9373644	96559466	C	T	309	2091	146	1816	0.12875	0.87125	0.502097261	1.838092805	7.00E-09
6	rs9390855	96553276	A	G	2091	309	1816	146	0.87125	0.12875	0.502097261	0.544042171	7.00E-09
6	rs9399756	96554687	A	G	309	2091	146	1816	0.12875	0.87125	0.502097261	1.838092805	7.00E-09
6	rs9399757	96556481	C	T	2091	309	1816	146	0.87125	0.12875	0.502097261	0.544042171	7.00E-09
6	rs9399758	96557119	C	T	309	2091	146	1816	0.12875	0.87125	0.502097261	1.838092805	7.00E-09
7	rs17340395	15771599	C	T	1742	658	1610	400	0.725833333	0.274166667	0.360833268	0.657743208	7.21E-09
18	rs71368937	51175275	A	G	2052	304	1783	143	0.870967742	0.129032258	0.313575015	0.541362872	7.36E-09
6	rs62416183	116081036	C	T	305	2035	392	1618	0.13034188	0.86965812	0.14699889	0.618625583	8.57E-09
1	rs12121630	50977279	A	G	286	2032	370	1606	0.123382226	0.876617774	0.062598145	0.610922537	8.65E-09
4	rs149486449	38852586	A	G	2123	191	1725	275	0.917458946	0.082541054	0.530084	1.771985735	8.93E-09
9	rs10959167	10559102	A	G	281	2091	128	1800	0.11846543	0.88153457	1	1.889795552	9.67E-09
1	rs61287776	38666444	A	G	154	2200	227	1739	0.065420561	0.934579439	0.797802683	0.536255507	1.05E-08
6	rs116630455	32601946	C	T	294	1992	385	1609	0.128608924	0.871391076	0.914767007	0.616812705	1.08E-08
19	rs2074064	58018618	A	G	2154	134	1725	207	0.941433566	0.058566434	0.820164097	1.928955224	1.12E-08
18	rs12457421	51176582	C	T	2046	296	1826	144	0.873612297	0.126387703	0.204450899	0.545099316	1.14E-08
7	rs73292262	15772269	A	C	1733	655	1597	399	0.725711893	0.274288107	0.332132543	0.661036199	1.17E-08
7	rs73680500	15772257	C	T	655	1733	399	1597	0.274288107	0.725711893	0.332132543	1.512776459	1.17E-08

6	rs115799091	31234569	C	T	726	1674	773	1237	0.3025	0.6975	0.966441938	0.69401902	1.21E-08
14	rs113363251	106023386	C	T	604	1734	679	1331	0.258340462	0.741659538	0.700610238	0.68280411	1.23E-08
6	rs9358916	26283185	A	G	2054	290	1632	374	0.876279863	0.123720137	0.698142476	1.623132184	1.26E-08
6	rs1130398	32629764	C	T	844	1548	879	1131	0.352842809	0.647157191	0.959635947	0.70152834	1.28E-08
6	rs9273505	32628420	C	T	844	1548	879	1131	0.352842809	0.647157191	0.959635947	0.70152834	1.28E-08
6	rs12176483	96571904	A	G	299	2085	141	1805	0.125419463	0.874580537	0.644049929	1.835790942	1.29E-08
4	rs28662935	20016275	A	G	384	2014	195	1743	0.160133445	0.839866555	0.789103023	1.704254832	1.32E-08
6	rs9377386	96567718	A	G	306	2094	146	1816	0.1275	0.8725	0.437425902	1.817639439	1.40E-08
6	rs12525187	26337537	A	G	303	2057	385	1625	0.128389831	0.871610169	0.442673334	0.621728782	1.42E-08
6	rs9386321	96572173	G	T	2085	299	1801	141	0.874580537	0.125419463	0.644049929	0.545934161	1.47E-08
3	rs35893577	46292944	C	T	1845	439	1450	524	0.807793345	0.192206655	0.659659843	1.518780928	1.51E-08
5	rs2451006	38358027	C	T	2060	228	1647	309	0.90034965	0.09965035	1	1.695107532	1.60E-08
7	rs143508017	52371108	C	T	2214	118	1802	190	0.949399657	0.050600343	0.35585365	1.978310352	1.63E-08
6	rs195506	116193934	A	C	287	2023	374	1628	0.124242424	0.875757576	0.719337485	0.617545288	1.65E-08
6	rs9366650	26335997	A	G	2058	304	1625	385	0.871295512	0.128704488	0.426712105	1.603906883	1.69E-08
1	rs1618566	164607346	A	G	401	1997	469	1519	0.167222686	0.832777314	0.095937627	0.650356131	1.69E-08
6	rs72844399	32634264	C	T	2162	158	1753	235	0.931896552	0.068103448	0.683865108	1.834359904	1.69E-08
11	rs75123752	35997294	C	T	2104	260	1624	332	0.89001692	0.10998308	0.593199172	1.654338765	1.70E-08
6	rs9390863	96559132	A	C	306	2092	147	1817	0.127606339	0.872393661	0.44005665	1.807995474	1.77E-08
6	rs9379844	26291527	A	G	2063	295	1632	376	0.874893978	0.125106022	0.603281259	1.611183117	1.78E-08
7	rs29696	41838649	C	T	342	2044	169	1767	0.143336127	0.856663873	0.639000461	1.749423916	1.83E-08
6	rs74775396	96551654	A	G	306	2094	147	1817	0.1275	0.8725	0.437425902	1.806268639	1.87E-08
22	rs135800	49948734	C	T	1991	405	1731	211	0.83096828	0.16903172	0.880816528	0.599241144	1.89E-08
18	rs12968161	51174711	C	T	2052	296	1825	145	0.873935264	0.126064736	0.451464289	0.550796001	1.91E-08
5	rs8185100	46250721	A	G	1125	1155	800	1166	0.493421053	0.506578947	0.346153855	1.419642857	1.97E-08
6	rs75668953	96500331	C	T	302	2084	144	1802	0.126571668	0.873428332	0.374974965	1.813433035	1.98E-08

6	rs13210271	96531782	A	C	2087	309	1820	150	0.871035058	0.128964942	0.507805663	0.556652086	1.99E-08
22	rs8140696	49949112	A	G	1995	405	1733	211	0.83125	0.16875	0.890028429	0.599752089	1.99E-08
6	rs4364486	96534389	C	T	309	2089	150	1820	0.128857381	0.871142619	0.504945727	1.794734323	2.10E-08
6	rs6571015	96534478	A	G	2089	309	1820	150	0.871142619	0.128857381	0.504945727	0.557185533	2.10E-08
22	rs135794	49947156	C	T	405	1995	211	1731	0.16875	0.83125	0.890028429	1.665431351	2.15E-08
22	rs135796	49947789	A	G	1995	405	1731	211	0.83125	0.16875	0.890028429	0.600445044	2.15E-08
22	rs135798	49948085	A	G	1995	405	1731	211	0.83125	0.16875	0.890028429	0.600445044	2.15E-08
22	rs135799	49948322	A	G	405	1995	211	1731	0.16875	0.83125	0.890028429	1.665431351	2.15E-08
22	rs135801	49948776	A	G	405	1995	211	1731	0.16875	0.83125	0.890028429	1.665431351	2.15E-08
11	rs7928683	42594849	A	C	334	2058	163	1765	0.139632107	0.860367893	1	1.757349741	2.23E-08
11	rs988605	42563831	C	T	2045	319	1758	154	0.865059222	0.134940778	1	0.56157075	2.28E-08
12	rs12580471	130704852	C	T	520	1864	587	1423	0.218120805	0.781879195	0.376115917	0.676276404	2.30E-08
22	rs135791	49946223	C	T	1995	405	1729	211	0.83125	0.16875	0.890028429	0.601139601	2.32E-08
6	rs75408081	96545305	G	T	2092	306	1818	148	0.872393661	0.127606339	0.44005665	0.556555002	2.36E-08
11	rs7114911	42589657	G	T	2060	334	1765	163	0.860484545	0.139515455	1	0.569591695	2.36E-08
11	rs1462683	42567773	G	T	2047	319	1758	154	0.865173288	0.134826712	1	0.562119964	2.41E-08
11	rs35456138	42559743	C	T	2047	319	1758	154	0.865173288	0.134826712	1	0.562119964	2.41E-08
11	rs35921631	42561389	C	T	319	2047	154	1758	0.134826712	0.865173288	1	1.778979692	2.41E-08
11	rs7103604	42597177	C	T	334	2062	163	1765	0.139398998	0.860601002	1	1.753940721	2.50E-08
6	rs9393700	26309380	A	G	2017	291	1632	378	0.873916811	0.126083189	0.195522747	1.605404791	2.52E-08
18	rs55826947	51178305	C	T	2051	297	1829	147	0.87350937	0.12649063	0.212860459	0.555025377	2.52E-08
7	rs2078899	22697325	A	G	2117	245	1840	110	0.896274344	0.103725656	0.574522413	0.516570541	2.54E-08
6	rs9358918	26286744	C	T	294	2058	374	1632	0.125	0.875	0.617148403	0.623376623	2.56E-08
6	rs66957194	32634263	G	T	2157	159	1753	235	0.93134715	0.06865285	0.659758198	1.818607455	2.58E-08
1	rs6658024	14900716	C	T	1009	1387	660	1292	0.421118531	0.578881469	0.409827728	1.42407638	2.60E-08
12	rs12580487	130704950	C	T	526	1874	572	1378	0.219166667	0.780833333	0.30026437	0.676190938	2.62E-08

6	rs9377335	96502789	C	T	302	2084	145	1803	0.126571668	0.873428332	0.374974965	1.801926004	2.64E-08
6	rs9404173	96502676	C	T	302	2084	145	1803	0.126571668	0.873428332	0.374974965	1.801926004	2.64E-08
6	rs13198397	116103759	C	T	2027	301	1564	372	0.870704467	0.129295533	0.11575997	1.601745248	2.66E-08
11	rs7112567	42599727	A	C	2062	334	1770	164	0.860601002	0.139398998	1	0.572022058	2.84E-08
18	rs78735241	51178691	A	G	295	2051	146	1828	0.125745951	0.874254049	0.188392242	1.800858919	2.86E-08
16	rs9931717	71813959	C	T	425	1889	503	1477	0.18366465	0.81633535	0.380629599	0.660647023	2.89E-08
11	rs12420480	42593504	A	G	2061	333	1765	163	0.860902256	0.139097744	1	0.571579512	2.93E-08
11	rs12421783	42590059	C	T	2061	333	1765	163	0.860902256	0.139097744	1	0.571579512	2.93E-08
11	rs58324933	42582945	A	G	2061	333	1765	163	0.860902256	0.139097744	1	0.571579512	2.93E-08
11	rs60587996	42593676	G	T	333	2061	163	1765	0.139097744	0.860902256	1	1.749537868	2.93E-08
11	rs7948264	42592249	C	T	2061	333	1765	163	0.860902256	0.139097744	1	0.571579512	2.93E-08
11	rs7948406	42592479	A	G	2061	333	1765	163	0.860902256	0.139097744	1	0.571579512	2.93E-08
6	rs114443705	31325317	C	T	2133	159	1762	238	0.930628272	0.069371728	1	1.812027499	2.94E-08
3	rs9827245	133144383	A	G	378	1980	456	1554	0.160305344	0.839694656	0.794766949	0.650598086	3.03E-08
2	rs17012046	32881819	A	G	2009	387	1717	197	0.838480801	0.161519199	0.708273829	0.595614007	3.03E-08
11	rs4310594	42594104	A	G	333	2063	163	1765	0.138981636	0.861018364	1	1.747841758	3.10E-08
11	rs7118124	42597329	A	G	2063	333	1765	163	0.861018364	0.138981636	1	0.572134174	3.10E-08
11	rs7118301	42597480	A	G	2063	333	1765	163	0.861018364	0.138981636	1	0.572134174	3.10E-08
11	rs7118467	42597333	A	G	333	2063	163	1765	0.138981636	0.861018364	1	1.747841758	3.10E-08
11	rs7949028	42583696	C	T	333	2063	163	1765	0.138981636	0.861018364	1	1.747841758	3.10E-08
11	rs80231181	42591876	A	G	2063	333	1765	163	0.861018364	0.138981636	1	0.572134174	3.10E-08
11	rs80268929	42596659	A	G	333	2063	163	1765	0.138981636	0.861018364	1	1.747841758	3.10E-08
17	rs8077846	68904487	A	G	1896	444	1437	507	0.81025641	0.18974359	0.490847414	1.506629803	3.12E-08
17	rs56889205	50910700	C	T	819	1581	504	1414	0.34125	0.65875	0.18843887	1.453352309	3.30E-08
17	rs9903784	50911254	A	G	819	1581	504	1414	0.34125	0.65875	0.18843887	1.453352309	3.30E-08
17	rs9905345	50910883	A	G	1581	819	1414	504	0.65875	0.34125	0.18843887	0.688064411	3.30E-08

17	rs9906552	50911407	A	G	1581	819	1414	504	0.65875	0.34125	0.18843887	0.688064411	3.30E-08
17	rs9911159	50912074	A	G	819	1581	504	1414	0.34125	0.65875	0.18843887	1.453352309	3.30E-08
7	rs28270	41834886	A	G	2059	341	1785	171	0.857916667	0.142083333	0.438019996	0.578442051	3.31E-08
14	rs10151594	51864477	A	G	303	2089	143	1773	0.126672241	0.873327759	0.334684901	1.798361045	3.37E-08
7	rs975347	45256232	C	T	2108	292	1786	136	0.878333333	0.121666667	1	0.549724647	3.46E-08
14	rs144577571	106033713	A	G	601	1691	674	1308	0.262216405	0.737783595	0.511841355	0.689729358	3.52E-08
14	rs149744714	106033620	C	T	601	1691	674	1308	0.262216405	0.737783595	0.511841355	0.689729358	3.52E-08
6	rs13210340	26326314	A	G	1994	352	1559	427	0.849957374	0.150042626	0.113771227	1.551544551	3.63E-08
10	rs3824636	127740245	G	T	2033	259	1661	345	0.886998255	0.113001745	0.25451865	1.630373385	3.65E-08
11	rs77384632	35903907	C	T	155	2135	234	1776	0.06768559	0.93231441	0.046328675	0.55101183	3.69E-08
17	rs8073953	50914336	C	T	1581	819	1412	504	0.65875	0.34125	0.18843887	0.689039006	3.73E-08
17	rs8078578	50914468	C	T	819	1581	504	1412	0.34125	0.65875	0.18843887	1.451296648	3.73E-08
11	rs2508584	63757446	C	T	193	2099	273	1717	0.084205934	0.915794066	1	0.578299103	3.73E-08
13	rs77529646	87203088	C	T	2115	165	1764	246	0.927631579	0.072368421	0.516519757	1.787569573	3.74E-08
14	rs61997108	104782996	A	C	168	2136	247	1763	0.072916667	0.927083333	0.300611656	0.561388346	3.78E-08
6	rs62394768	26326033	C	T	353	1995	431	1573	0.150340716	0.849659284	0.107972263	0.645778018	3.83E-08
1	rs3850874	51063131	C	T	2056	300	1592	370	0.872665535	0.127334465	0.052350603	1.59279732	3.96E-08
6	rs4473904	32635590	A	G	2123	167	1747	245	0.927074236	0.072925764	1	1.782816736	4.03E-08
12	rs77649070	66279618	A	G	178	2196	251	1759	0.074978939	0.925021061	0.363124	0.568041132	4.03E-08
7	rs29694	41839311	A	G	340	2060	171	1785	0.141666667	0.858333333	0.56615507	1.722875149	4.10E-08
7	rs29695	41838814	C	T	2060	340	1785	171	0.858333333	0.141666667	0.56615507	0.580425111	4.10E-08
7	rs29697	41834611	C	T	340	2060	171	1785	0.141666667	0.858333333	0.56615507	1.722875149	4.10E-08
6	rs9399754	96553006	A	G	2094	302	1817	147	0.873956594	0.126043406	0.363120987	0.56096032	4.16E-08
6	rs186237401	96561603	A	G	300	2090	146	1816	0.125523013	0.874476987	0.481397794	1.785409976	4.22E-08
22	rs135821	49956008	G	T	398	1988	210	1732	0.16680637	0.83319363	1	1.65118329	4.24E-08
17	rs7222836	50907828	A	G	1581	819	1414	506	0.65875	0.34125	0.18843887	0.690794825	4.49E-08

17	rs8079528	50908070	A	G	1581	819	1414	506	0.65875	0.34125	0.18843887	0.690794825	4.49E-08
4	rs10008797	168075537	A	G	125	2199	193	1773	0.053786575	0.946213425	0.070454085	0.52219921	4.49E-08
6	rs9273794	32629482	A	G	385	2003	447	1521	0.161222781	0.838777219	0.74247257	0.654035725	4.53E-08
9	rs145713521	99818281	A	G	140	2152	212	1754	0.061082024	0.938917976	0.528315677	0.538244371	4.58E-08
9	rs145752554	99818102	A	G	140	2152	212	1754	0.061082024	0.938917976	0.528315677	0.538244371	4.58E-08
7	rs12532134	104922603	A	G	143	2193	216	1794	0.061215753	0.938784247	0.567445287	0.541584334	4.59E-08
22	rs8140866	49949306	A	C	1989	399	1733	211	0.832914573	0.167085427	1	0.606940028	4.60E-08
7	rs75737264	52383865	C	T	124	2272	188	1794	0.051752922	0.948247078	0.447113247	0.520808361	4.80E-08
6	rs2893843	26338697	A	G	298	2046	372	1608	0.127133106	0.872866894	0.520212304	0.629584082	4.83E-08
1	rs2102188	50840015	A	G	2028	298	1627	379	0.871883061	0.128116939	0.711166497	1.585270375	4.91E-08

Supplementary Table S2. Association results in GWAS stage for Nephrolithiasis susceptibility loci identified in populations of European or Japanese populations.

CHR	SNP	BP	Allele1	Allele2	Control Allele1	Control Allele2	Case Allele1	Case Allele2	ControlAllele1 Freq	ControlAllele2 Freq	HWE	OR	P
1	rs10917002	21836340	C	T	1700	692	1362	588	0.710702341	0.289297659	0.95480138	1.06057905	0.397292849
1	rs1256328	21896767	C	T	1878	514	1582	428	0.785117057	0.214882943	0.32648336	0.98848426	0.904440105
2	rs1260326	27730940	C	T	1105	1293	885	1125	0.460800667	0.539199333	0.47886337	1.0863581	0.182871963
2	rs780093	27742603	C	T	1118	1242	916	1094	0.473728814	0.526271186	0.22844239	1.07508315	0.246459076
4	rs1481012	89039082	A	G	1615	741	1353	657	0.685483871	0.314516129	0.50165332	1.05833191	0.401350728
5	rs11746443	176798306	A	G	410	1892	383	1627	0.178105995	0.821894005	0.42193507	0.92055874	0.311259407
5	rs12654812	176794191	A	G	686	1714	593	1417	0.285833333	0.714166667	0.23159627	0.95637553	0.524370905
6	rs1544935	39124448	G	T	371	2027	284	1658	0.15471226	0.84528774	0.40127111	1.06852908	0.463838187
6	rs3798519	50788778	A	C	1876	524	1439	479	0.781666667	0.218333333	0.02045497	1.19172559	0.016757993*
7	rs6975977	30917831	A	G	245	2137	178	1744	0.102854744	0.897145256	0.77739908	1.12328004	0.284310833
16	rs77924615	20392332	A	G	421	1975	420	1590	0.175709516	0.824290484	0.05773984	0.80698011	0.005808825*
20	rs13041834	52703284	C	T	579	1777	427	1561	0.245755518	0.754244482	1	1.1911492	0.017580621*

Supplementary Table S3. The result of association analysis of nephrolithiasis in the replication stage.

SNP	Chr	Position	Region	Effect Allele	Non effect Allele	Gene	Case MAF	Control MAF	CHISQ	OR	SE	95% CI	P
rs9827245	3	133144383	3q22.1	A	G	BFSP2	0.2292	0.1966	3.999	1.215	0.09748	1.004-1.471	0.04552
rs3736194	4	186566987	4q35.1	G	A	SORBS2	0.1667	0.2366	17.31	0.6453	0.1058	0.5244-0.794	3.18E-05*
rs11132928	4	173052396	4q34.1	A	C	GALNTL6	0.1075	0.1354	4.228	0.7688	0.1281	0.5981-0.9883	0.03976
rs356450	5	139055009	5q31.2	G	A	CXXC5	0.1112	0.07164	12.6	1.622	0.1373	1.239-2.123	0.000387*
rs9264533	6	31234494	6p21.33	T	C	HCG27, HLA-C	0.1891	0.1589	3.942	1.234	0.1062	1.002-1.52	0.0471
rs141362726	6	96570804	6q16.1	T	C	FUT9	0.06893	0.09226	4.194	0.7283	0.1553	0.5372-0.9875	0.04057
rs2078899	7	22697325	7p15.3	G	A	LOC401312	0.0591	0.08746	6.553	0.6554	0.1661	0.4733-0.9076	0.01047
rs975347	7	45256232	7p13	T	C	RAMP3, ADCY1	0.07665	0.1039	5.105	0.716	0.1484	0.5353-0.9577	0.02386
rs29695	7	41838814	7p14.1	T	C	INHBA-AS1, GLI3	0.1016	0.1322	5.2	0.7426	0.1309	0.5746-0.9597	0.02259
rs10959167	9	10559102	9p23	A	G	PTPRD	0.07126	0.1002	5.954	0.689	0.1534	0.5101-0.9307	0.01469
rs17155703	10	14701678	10p13	C	T	FAM107B	0.05012	0.0873	11.82	0.5516	0.1752	0.3912-0.7776	0.000585*
rs12218684	10	130365816	10q26.2	C	T	LINC01163, MGMT	0.07944	0.1086	5.693	0.7081	0.1453	0.5327-0.9413	0.01703
rs10151594	14	51864477	14q22.1	A	G	LINC00640, FRMD6	0.0808	0.1254	11.91	0.6131	0.1429	0.4634-0.8113	0.00056*
rs8079079	17	68901561	17q24.3	A	G	KCNJ2, CASC17	0.1856	0.2212	4.629	0.8028	0.1022	0.6571-0.9809	0.03143
rs55826947	18	51178305	18q21.2	T	C	LOC102724651, MBD2	0.09908	0.1391	8.745	0.6807	0.1307	0.5269-0.8794	0.003105*
